# Event-Related-Potential (ERP) Correlates of Performance Monitoring in Adults With Attention-Deficit Hyperactivity Disorder (ADHD)

**DOI:** 10.3389/fpsyg.2018.00485

**Published:** 2018-04-11

**Authors:** Lynn Marquardt, Heike Eichele, Astri J. Lundervold, Jan Haavik, Tom Eichele

**Affiliations:** ^1^K.G. Jebsen Centre for Research on Neuropsychiatric Disorders, Department of Biomedicine, University of Bergen, Bergen, Norway; ^2^Department of Biological and Medical Psychology, University of Bergen, Bergen, Norway; ^3^Division of Psychiatry, Haukeland University Hospital, Bergen, Norway; ^4^Section for Neurophysiology, Department of Neurology, Haukeland University Hospital, Bergen, Norway

**Keywords:** event-related potentials, ADHD, P3, ERN, Pe, performance monitoring

## Abstract

**Introduction:** Attention-deficit hyperactivity disorder (ADHD) is one of the most frequent neurodevelopmental disorders in children and tends to persist into adulthood. Evidence from neuropsychological, neuroimaging, and electrophysiological studies indicates that alterations of error processing are core symptoms in children and adolescents with ADHD. To test whether adults with ADHD show persisting deficits and compensatory processes, we investigated performance monitoring during stimulus-evaluation and response-selection, with a focus on errors, as well as within-group correlations with symptom scores.

**Methods:** Fifty-five participants (27 ADHD and 28 controls) aged 19–55 years performed a modified flanker task during EEG recording with 64 electrodes, and the ADHD and control groups were compared on measures of behavioral task performance, event-related potentials of performance monitoring (N2, P3), and error processing (ERN, Pe). Adult ADHD Self-Report Scale (ASRS) was used to assess ADHD symptom load.

**Results:** Adults with ADHD showed higher error rates in incompatible trials, and these error rates correlated positively with the ASRS scores. Also, we observed lower P3 amplitudes in incompatible trials, which were inversely correlated with symptom load in the ADHD group. Adults with ADHD also displayed reduced error-related ERN and Pe amplitudes. There were no significant differences in reaction time (RT) and RT variability between the two groups.

**Conclusion:** Our findings show deviations of electrophysiological measures, suggesting reduced effortful engagement of attentional and error-monitoring processes in adults with ADHD. Associations between ADHD symptom scores, event-related potential amplitudes, and poorer task performance in the ADHD group further support this notion.

## Introduction

Attention-deficit hyperactivity disorder (ADHD) is a frequent neurodevelopmental disorder in children, and recent research has shown that ADHD symptoms persist into adulthood in about 15–65% of childhood cases (Faraone et al., 2006), with a rate depending on the diagnostic criteria used to define the disorder. Symptoms in adults include increased impulsiveness and activity levels, as well as a feeling of inner restlessness. Even more commonly, adults with ADHD describe difficulties to sustain and direct attention toward relevant stimuli and, at the same time, ignoring other information that distracts them from focusing on relevant items (Bush, 2010; Hasler et al., 2016). These symptoms can lead to poorer neuropsychological performance, emotional reactivity, and motivation (Haavik et al., 2010), and may thus interfere with academic, occupational, and social functioning (Halleland et al., 2012). Recent neuroimaging studies of individuals with ADHD indicate that multiple brain regions are involved in the pathophysiology of ADHD. Converging evidence from these studies point at alterations in fronto-striatal networks (for an overview, see Bush, 2010) which are essential for regulation of attention and behavior, and influence inhibitory control (Durston et al., 2002, 2003). The present study includes scalp electrophysiological data. Although these data are not optimal to localize the underlying brain structure, they provide good temporal resolution and chronometric information about different brain functions.

The ability to dynamically adjust attention and behavior to situational demands, hence to monitor performance, is a crucial part of adequate daily functioning (Ullsperger and von Cramon, 2006; Ullsperger et al., 2014a), and requires a set of processing functions that are localized to a broad network of brain areas encompassing fronto-striatal regions. The performance monitoring system mediates adaptation of goal-directed behavior and provides neural signals for adjustments of responses after errors and, more generally, whenever decisions are risky or uncertain. Event-related electrical activity that corresponds to these functions during stimulus processing from about 250 ms after onset includes the N2 and P3. The N2 component is thought to represent detection of and adaptation to uncertainty, mismatch, and conflict, while the following P3 response relates to recruitment and resource allocation necessary for task performance. Response-locked activity is generated mainly in the medial frontal cortex and includes the error-related negativity (ERN) and error positivity (Pe). The ERN occurs about 50–100 ms after incorrect responses and has a fronto-central scalp distribution. It is thought to reflect post-response conflict between executed and competing response tendencies and suggests a rapid internal detection mechanism (Yeung et al., 2004; Jocham and Ullsperger, 2009; Ullsperger et al., 2014a). Following the ERN, the Pe occurs about 300–500 ms after an error and has a centro-parietal distribution. While it is uncertain if the ERN is affected by the awareness of making an error, the Pe is a marker for error awareness, with a more positive deflection after perceived errors compared to undetected errors (Ullsperger, 2006; Ullsperger et al., 2014a,b).

Several studies have shown impairments in performance monitoring tasks in children and adults with ADHD, and concomitant changes in the fronto-striatal network. Considering electrophysiology, some studies point to smaller ERN amplitudes in children with ADHD (Liotti et al., 2005; van Meel et al., 2007; Groen et al., 2008; Senderecka et al., 2012), as well as in college-aged adolescents (Chang et al., 2009) and adults (Herrmann et al., 2010). However, other authors did not find significant differences between participants with ADHD and controls (Wiersema et al., 2005; Jonkman et al., 2007; Groom et al., 2010; Van De Voorde et al., 2010; Shen et al., 2011). Correspondingly, lower Pe amplitudes were found in individuals with ADHD compared to controls (Groen et al., 2008; Herrmann et al., 2009; Zhang et al., 2009; Groom et al., 2010; Van De Voorde et al., 2010; Shen et al., 2011), however not consistently (Albrecht et al., 2008; Chang et al., 2009; Wild-Wall et al., 2009), possibly due to heterogeneity between clinical samples or methodological differences. McLoughlin et al. (2009) did not find differences in Pe, but ERN and N2 were reduced in adults with ADHD in a flanker task. Wild-Wall et al. (2009) did also find a significantly lower N2 amplitude in ADHD compared to controls, but no effect for Pe, in a flanker task that had a No-Go condition.

A recent small-scale meta-analysis on error monitoring in adults with ADHD including seven electrophysiology studies with both flanker and Go/NoGo tasks concluded that Pe-differences in flanker tasks were not robust, but that Pe amplitudes were consistently lower in individuals with ADHD in Go/NoGo tasks. ERN amplitudes were significantly reduced in adults with ADHD in both types of task. Behaviorally, higher error rates and overall slower response times (RTs) were present in the ADHD group (Geburek et al., 2013).

While research into performance monitoring commonly considers response-related EEG/ERP components, the initial stimulus processing and evaluation leading up to a response is equally relevant. The N2 and P3 are the main components in the post-stimulus interval that indicate stimulus evaluation, response selection, and sensitivity to manipulation of stimulus/response conflict. ERP studies in children with ADHD show reduced component amplitudes also during attentional processing in the P3 range (Wiersema et al., 2006; Johnstone et al., 2013; Heinrich et al., 2014; Cheung et al., 2016; Eichele et al., 2016). In a selective meta-analysis of eight studies including Go/NoGo paradigms, adults with ADHD showed a moderate decrease of P3 (Szuromi et al., 2011). Additionally, studies including college-age students (Woltering et al., 2013) or adults (Fisher et al., 2011) found no difference in N2 amplitudes, but a reduced P3 in the ADHD group. Smaller P3 amplitudes in individuals with ADHD were also present in working memory tasks (Wiersema et al., 2009; Kim et al., 2014). Considering other P3-eliciting experiments more broadly, smaller P3 amplitudes in adults with ADHD were also seen in an auditory oddball task (Itagaki et al., 2011).

The available literature is not definitive regarding possible electrophysiological impairments in people with ADHD, and few studies have investigated the relationship between electrophysiological measures and symptom severity. Herrmann et al. (2010) compared individuals with low- and high-ADHD symptom scores in a non-clinical population, and observed lower Pe in the group with higher scores. Similarly, Wiersema et al. (2009) reported negative correlations between Pe amplitudes and ADHD symptoms as well as between ADHD symptoms and P3 amplitudes in the NoGo condition of a Go-NoGo task (Wiersema and Roeyers, 2009) in individuals with ADHD. Considering the conflicting results, it is important to investigate alterations in the fronto-parietal network and the relation to ADHD symptomatology.

We therefore tested a sample of adults with ADHD with a broad age range (19–55 years) in a speeded variant flanker task. Their behavioral performances, and stimulus and response processing were assessed by an ERP paradigm, and their results were compared to results in a control group and correlated with an ADHD symptom score.

It is worth noting that intra-individual variability is generally higher in ADHD than in control populations (Castellanos et al., 2005), and that manipulations in task difficulty, speed, and positive feedback can ameliorate performance differences in individuals with ADHD (Andreou et al., 2007; Kuntsi et al., 2009; Kuntsi et al., 2013; Cheung et al., 2017). In order to minimize variability and ensure optimal performance for both groups with time on task, we therefore employed a speeded self-paced task with feedback on errors and reaction time (RT) slowing in order to minimize behavioral influence on electrophysiological components.

Age is another important element of variation that will be statistically controlled for in this report, both because ADHD symptoms change across the lifespan (Seidman, 2006), and because performance monitoring also varies as a function of aging (Larson et al., 2016).

At the outset, we hypothesized that adults with ADHD would display reduced P3, ERN, and Pe components, as well as slower RT and reduced accuracy (ACC) for participants with ADHD compared to controls. We also hypothesized that amplitude reductions relate to higher symptom load within the patient group.

## Materials and Methods

### Participants in the Main Study: ADHD in Norwegian Adults

The participants of the present study were recruited from a larger Norwegian project on adults with ADHD. In the main study, all participants with ADHD were formally diagnosed according to national guidelines on referral from a National registry of adults in Norway and from psychologists and psychiatrists nation-wide. These guidelines were developed by an expert committee according to current criteria in the International Classification of Diseases – Tenth Revision (ICD-10; World Health Organization, 1992) with the allowance for the diagnosis of the inattentive subtype according to the Diagnostic and Statistical Manual of Mental Disorders, fourth edition (DSM-IV; American Psychiatric Association, 1994).

A sample of individuals in the same age range as the patients was recruited from the general population in Norway through the Medical Birth Registry of Norway (MBRN) to serve as control group. The project was approved by the Regional Committee for Medical and Health Research Ethics of Western Norway [Institutional Review Board (IRB) 00001872]. Further details concerning the main study are described in previous publications (Halmoy et al., 2009, 2010).

### Participants in the Present Study

A subsample from the main study living in or around the Bergen municipality was invited to take part in an extended clinical examination including a set of neuropsychological tests and a psychiatric interview (*n* = 80 with ADHD and *n* = 80 controls) (see also Halleland et al., 2012). The subgroup included in the present study participated in a follow-up study including electrophysiological recordings (*N* = 63, 30 males). The ADHD group included 31 adults (age range = 19–55 years) and the control group 32 adults (age range = 19–45 years). Ethical approval for this study was obtained from the Regional Committee for Medical Research Ethics, West-Norway, and written informed consent in accordance with the Declaration of Helsinki was obtained from all participants. Full-scale intelligence quotient (FSIQ) was assessed by two subtests (Matrix Reasoning and Vocabulary) from the Wechsler-Abbreviated Scale of Intelligence (Wechsler, 1999). Forty-eight participants were right-handed, and groups did not differ regarding FSIQ, sex, age, or handedness. ADHD symptoms were determined by the World Health Organization’s Adult ADHD Self-Report Scale (ASRS) (Kessler et al., 2005; **Table 1**), shown to be a useful tool to identify adult ADHD (Kessler et al., 2007). ASRS is designed to measure current ADHD symptoms, representing the 18 DSM-IV symptoms of ADHD. The items are scored on a 5-point Likert scale, with nine items reflecting the hyperactive-impulsive (score range 0–36) and nine items the inattentive presentation (score range 0–36). Here, we include the total scores across the two subscales and the inattention subscale, where a higher score indicates higher severity level. The inattentive subscore was assumed to relate to ERP measures of attention function. Correlations between symptom severity and ERP components have been documented in studies using ASRS (Wiersema et al., 2009; Herrmann et al., 2010) as well as other rating scales, like Conners’ Adult ADHD Rating Scale (Cross-Villasana et al., 2015; Wiegand et al., 2016). The following self-reported psychiatric comorbidities were present among the participants: anxiety/depression (ADHD *N* = 13, control *N* = 5), autism spectrum disorders (ADHD *N* = 1, controls *N* = 2), bipolar disorder (ADHD *N* = 2), alcohol-related problems (ADHD *N* = 2), drug-related problems (ADHD *N* = 2), treatment for other mental health problems (ADHD *N* = 8), and eating disorders (ADHD *N* = 4). Participants taking stimulants (ADHD *N* = 14) were asked to refrain from medication 48 h prior to investigation. Other types of medication (antihistamines *N* = 7, melatonin *N* = 1, antihypertensive *N* = 2, contraception *N* = 3, antidiabetics *N* = 1, asthma medication *N* = 3, statins *N* = 1, thyroxines *N* = 1) were taken as prescribed.

**Table 1:** Sample characteristics.

Sample statistics			
	**ADHD (*n* = 27)**	**Controls (*n* = 28)**	**Statistics**
	**Mean ± *SD***	**Mean ± *SD***	
FSIQ	111.25 ± 11	115.18 ± 9	*t* = -1.44, n.s.
Age (years)	35.32 ± 8.8	33.37 ± 7.0	*t* = 0.890, n.s.
Gender (% male)	59.26	42.86	χ^2^ = 1.48, n.s.
Handedness (% right handed)	85.19	90.32	χ^2^ = 0.208, n.s.
ASRS total scores (0–72)	42.78 ± 13	17.54 ± 7	*t* = 8.78, *p* < 0.001, *d* = 2.46
ASRS inattentive scores (0–36)	50.56 ± 20	12.00 ± 11	*t* = 8.96, *p* < 0.001, *d* = 2.50

*ADHD, attention-deficit/hyperactivity disorder; FSIQ, full-scale intelligence quotient; n.s., not significant; ASRS, adult ADHD self-reporting scale; χ^*2*^, chi-square; SD, standard deviation.*

### Experimental Design

After verbal instructions and a training session, participants performed a modified Eriksen flanker task implemented in E-prime 2 (Psychology Software Tools, Inc., Pittsburgh, PA, United States).

Participants were instructed to fixate a dot in the center of the PC screen. The dot was present in a fixed inter-trial interval of 800 ms without jitter. Trials began with six horizontal flanking arrows below the fixation dot that were shown for 100 ms and then followed by the appearance of a center target arrow that either pointed in the same direction as the flanking arrows ( < < < < < < < or > > > > > > > ), or in the opposite direction, ( < < < > < < < or > > > < > > > ), yielding compatible and incompatible trials, respectively. Participants were asked to respond as fast and accurate as possible with a mouse button click with their preferred hand in same direction as the target arrow. Trials remained on screen until a response button press was registered. Simple feedback was given on erroneous trials “x,” or on trials with RTs extending an adaptive threshold “!,” i.e., when RTs slower than the cumulative mean RT+1.5 standard deviations. The total trial interval was therefore partially self-paced around 1500 ms, i.e., 900 ms plus the individual RTs. Each participant completed 520 trials in a randomized sequence divided into two blocks with a short break in between. Overall, probability for right/left as well as compatible/incompatible was kept as 0.5.

### EEG Acquisition and Processing

Recordings took place in an electro-magnetically shielded chamber (Rainford, Wigan, United Kingdom). A 64-channel equidistant electrode cap with Ag/AgCl electrodes (BrainCap-MR3 64Ch from EASYCAP GmbH, Herrsching, Germany) was used. Data were sampled at 1000 Hz frequency with Brain Amp amplifiers (BrainProducts, Munich, Germany). The recording reference was placed at Cz, with a ground placed at approximately AFz. The data were offline re-referenced to the common average. Electrocardiogram was recorded from an additional channel. Impedances were kept below 10 kΩ. EEG data were pre-processed with MATLAB (MathWorks, Natick, MA, United States) with the EEGLAB toolbox (Delorme and Makeig, 2004) and in-house scripts. The continuous EEG data were resampled to 500 Hz and filtered from 0.5 to 40 Hz.

### Averaging and Data Extraction

After visual inspection to rule out EEG abnormalities, and pervasive signal artifacts, we performed automatic artifact rejection in order to denoise the data prior to independent component analysis (ICA). EEG epochs were detrended, and we computed for each channel the root mean-squared signal, as well as its differential, the standard deviation, skewness, kurtosis of the time series, and dynamic range of the power spectrum. These measures were normalized to unit variance, and epochs falling within ±1 standard deviation were retained for further analysis, concatenated, and subjected to temporal ICA using Infomax (Bell and Sejnowski, 1995). In order to identify and remove contributions from eye movements and blinks, a template correlation was used (Viola et al., 2009). Hereafter, sources were sorted on their spatial and temporal statistics and those contributing to the event-related responses were identified and retained (Wessel and Ullsperger, 2011). Stimulus-locked ERP was generated from -0.5 to +1 s from all stimulus events. Response-locked epochs were segmented -1 to +0.5 s around the button press. The pre-stimulus period served as baseline for both segments. Before re-averaging, epochs with residual artifacts were identified and removed using absolute amplitude and statistical thresholds. The number of rejected trials out of 520 varied between 15 and 107. On average, 59 trials in ADHD and 61 in controls were excluded from averaging. The average ERP was extracted from the data for stimulus-locked and response-locked ERP. After reviewing grand average ERP across all participants and conditions, we defined a region of interest from a five-electrode cluster (θ = 23, φ = 90; θ = 23, φ = 30; θ = 46, φ = 74; θ = -23, φ = -30; θ = -46, φ = -74) around FCz for further analysis based on the voltage maximum of the scalp topography.

**FIGURE 1:**
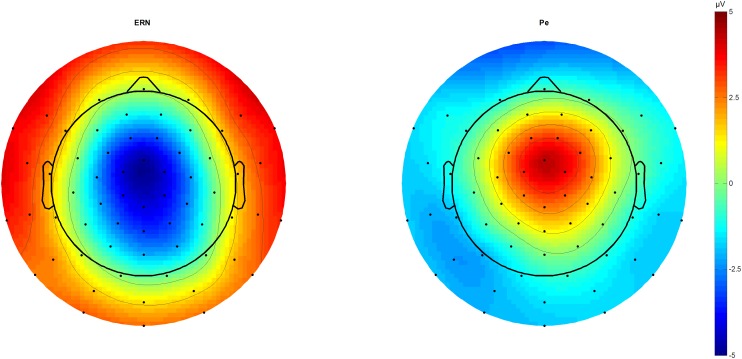
ERN and Pe topographic plots. Topographic plots of the amplitude difference between correct and incorrect outcomes at 46 (ERN) and 212 ms (Pe) post-response. Scaling from –5 to 5 μV. Voltage maximum is at fronto-central midline sites that were used for further component analysis as a region of interest average. ERN, error-related negativity; Pe, error positivity.

We identified N2 (340 ms) and P3 (440 ms) in the stimulus-locked grand averages and extracted averaged peaks from 40 ms long-time windows centered on the peak latency. In response-locked traces, peak extraction of the ERN was done by peak-to-peak analysis for 20–60 ms subtracted from the immediately preceding motor positivity (-40 – 0 ms), while the Pe was estimated from 180 to 220 ms post-response (**Figure 1**).

Response times and response ACC averages were generated for all possible outcomes for each participant. Premature responses faster than 200 ms and slow responses longer than 2000 ms were not considered in the ERP and RT averages, and rates of early commissions and omitted responses were recorded. Intra-individual RT variability within the bounded distribution was estimated as the standard deviation.

### Statistics

**FIGURE 2:**
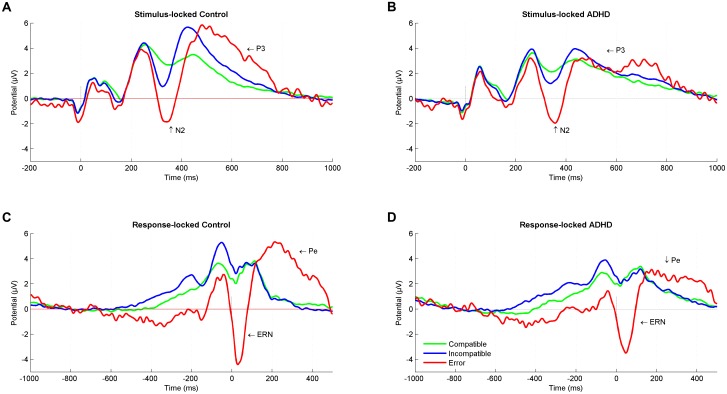
Stimulus-locked and response-locked event-related potentials (ERPs). Stimulus-locked Grand average ERP at a central region of interest for compatible (green), incompatible (blue), and error (red) trials in **(A)** control participants and **(B)** ADHD participants. Response-locked Grand average ERP for compatible (green), incompatible (blue), and error (red) trials in **(C)** control participants and **(D)** ADHD participants. ADHD, attention-deficit/hyperactivity disorder.

Statistics were computed in Matlab and Statistica (StatSoft, Tulsa, OK, United States). To address our main question, we estimated the compatibility effects and tested for differences between the groups. Compatibility effects refer to the difference between compatible and incompatible conditions, and were investigated for the behavioral measures RTs, ACC, and RT variability (sdRT), as well as the stimulus-locked ERPs N2 and P3 with repeated-measure analyses of variance (ANOVA) with compatibility as within-subject factor. We further investigated group differences of the error-specific response-locked ERPs ERN and Pe with correctness as within-subject factor and diagnosis as between-subject factor (**Figure 2**). To control for possible influence of age, we also included age as continuous predictor for the behavioral data, and additionally RT for the ERP components. All statistical tests were considered significant at *p* < 0.05. Relevant interaction effects were followed up with *post hoc* tests. Further, significant results were followed up by Pearson’s correlations to investigate associations between neurophysiological data and ADHD symptoms in the ADHD group. The effect-size indicator partial eta-squared (${\text{η}}_{\text{p}}^{2}$) is reported for each significant/trend-significant statistical comparison as a measure of the strength of the effect, with ${\text{η}}_{\text{p}}^{2}$ of 0.01 representing a small effect, ${\text{η}}_{\text{p}}^{2}$ = 0.06 a medium effect, and ${\text{η}}_{\text{p}}^{2}$ = 0.14 a large effect. For *t*-tests, Cohen’s *d* is shown as a measure for effect size, 0.20 being a small effect, 0.50 a medium, and from 0.80 being a large effect (Cohen, 1988). From the initial sample of 63 participants, a total of eight were excluded from further analysis. Three participants (ADHD *N* = 2, control *N* = 1) with excessive error rates >25% were excluded from the analyses, another (ADHD *N* = 1) due to lack of incompatible errors in the flanker task. One participant had to be excluded due to a technical error during EEG recording (ADHD *N* = 1). Three participants from the control sample had cutoff scores above the threshold for probable ADHD on the ASRS and were therefore also excluded. The remaining sample consisted of 55 participants, with 27 ADHD and 28 control participants, 28 males and 27 females.

## Results

Means of RTs, percentage of errors, as well as means of ERP amplitudes are shown in **Table 2**.

**Table 2:** Means of behavioral performance and ERP measures.

Means of behavioral performance and ERP amplitudes		

	**ADHD**	**Controls**
	**Mean ± *SD***	**Mean ± *SD***
**Behavioral performance**		
**Erroneous responses (%)**		
Compatible	2.60 ± 2.45	1.81 ± 2.07
Incompatible	12.91 ± 7.54	9.71 ± 6.68
**Reaction time (ms)**		
Compatible	444.07 ± 65.91	428.55 ± 79.34
Incompatible	558.57 ± 94.02	523.57 ± 90.95
**Reaction time variability (ms)**		
Compatible	135.40 ± 65.85	113.04 ± 52.43
Incompatible	148.20 ± 89.75	112.90 ± 60.80
**Stimulus-locked ERP amplitudes**		
**N2 (μV)**		
Compatible	2.18 ± 2.17	2.75 ± 2.23
Incompatible	1.31 ± 2.08	1.42 ± 2.45
**P3 (μV**)		
Compatible	3.08 ± 2.95	3.42 ± 1.91
Incompatible	3.88 ± 3.30	5.52 ± 2.41
**Response-locked ERP amplitudes**		
**ERN (μV)**		
Correct responses	-1.03 ± 1.20	-1.36 ± 1.32
Erroneous responses	-4.23 ± 2.78	-6.38 ± 3.73
**Pe (μV)**		
Correct responses	1.97 ± 1.85	0.99 ± 1.57
Erroneous responses	3.22 ± 3.46	5.35 ± 6.10

*ERP, event-related potentials; ADHD, attention-deficit/hyperactivity disorder; SD, standard deviation; ERN, error-related negativity; Pe, error positivity.*

### Behavioral Performance

#### Accuracy

**FIGURE 3:**
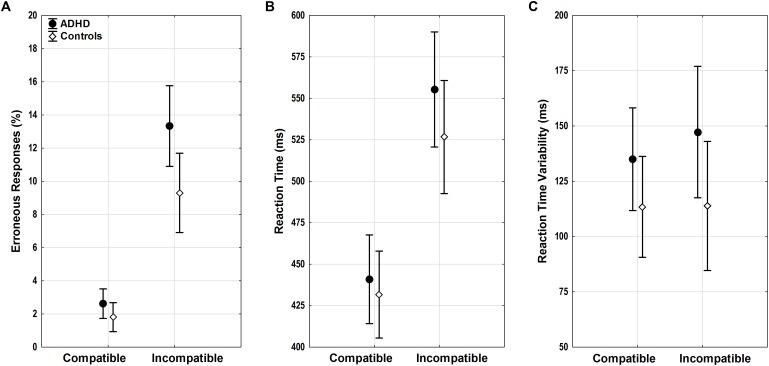
Flanker task performance. **(A)** Erroneous responses, **(B)** reaction time (RT), and **(C)** RT variability with means and 95% confidence intervals are displayed for compatible and incompatible trials in adults with ADHD (black) and control adults (white). ADHD, attention-deficit/hyperactivity disorder.

Both groups showed a typical flanker compatibility effect with more errors in incompatible trials (compatibility *F*_1,52_ = 37.39, *p* < 0.001, ${\text{η}}_{\text{p}}^{2}$ = 0.42). Error rates differed between groups (diagnosis *F*_1,52_ = 6.54, *p* = 0.01, ${\text{η}}_{\text{p}}^{2}$ = 0.11) (**Figure 3A**), and adults with ADHD showed a trend toward diverging effects of compatibility (compatibility ^∗^ diagnosis *F*_1,52_ = 3.43, *p* = 0.07, ${\text{η}}_{\text{p}}^{2}$ = 0.07) with more errors in incompatible trials than controls (*p* = 0.01). ACC increased with age (age *F*_1,52_ = 13.75, *p* < 0.001, ${\text{η}}_{\text{p}}^{2}$ = 0.21), with lower error rates particularly in incompatible trials in older adults with ADHD (compatibility ^∗^ age *F*_1,52_ = 14.79, *p* < 0.001, ${\text{η}}_{\text{p}}^{2}$ = 0.22, *r* = -0.62).

#### Reaction Times of Correct Responses

Similar flanker compatibility effects were seen for RTs with slower RTs in incompatible trials (compatibility *F*_1,52_ = 20.66, *p* < 0.001, ${\text{η}}_{\text{p}}^{2}$ = 0.28). RTs increased with age (age *F*_1,52_ = 6.01, *p* = 0.02, ${\text{η}}_{\text{p}}^{2}$= 0.1) regardless of compatibility (compatibility ^∗^ age *F*_1,52_ < 0.001, n.s.). While RTs did not differ between groups overall (diagnosis *F*_1,52_ = 0.81, n.s.) (**Figure 3B**), there was a trend toward larger RT increase under conflict in participants with ADHD when compared with controls (compatibility ^∗^ diagnosis *F*_1,52_ = 3.53, *p* = 0.07, ${\text{η}}_{\text{p}}^{2}$ = 0.06), however, without significant differences in the relevant follow-up *post hoc* tests.

#### Reaction Time Variability of Correct Responses

Reaction time variability did not show significant effects of compatibility, age, diagnosis, or any significant interactions (all *F* < 2.47, *p* > 0.1) (**Figure 3C**).

### Stimulus-Locked ERPs

#### N2

**FIGURE 4:**
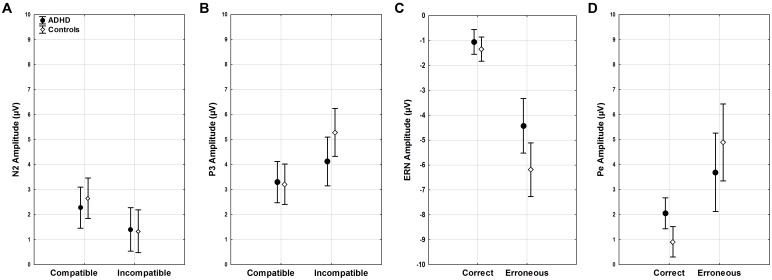
ERP amplitudes. Stimulus-locked ERP mean amplitudes for **(A)** N2 and **(B)** P3 in compatible and incompatible trials by group. Response-locked ERP mean amplitudes for **(C)** ERN and **(D)** Pe for correct and erroneous responses by group.

Amplitudes of N2 did not show differences for diagnosis, age, compatibility, or their interactions (all *F* < 3.45, *p* > 0.07). (**Figure 4A**). However, a trend toward more enhanced N2 amplitudes was seen with slower RTs (*F*_1,51_ = 3.82, *p* = 0.06).

#### P3

P3 amplitudes were smaller in younger than older participants (age *F*_1,51_ = 5.06, *p* < 0.03, ${\text{η}}_{\text{p}}^{2}$= 0.09) and with faster RTs (RT *F*_1,51_ = 9.87, *p* < 0.01, ${\text{η}}_{\text{p}}^{2}$ = 0.16). While both groups showed higher P3 amplitudes in incompatible than in compatible trials (diagnosis *F*_1,52_ = 0.81, n.s.), the compatibility effect was more pronounced in the controls (compatibility ^∗^ diagnosis *F*_1,51_ = 9.49, *p* = 0.003, ${\text{η}}_{\text{p}}^{2}$ = 0.16), with greater differences between compatible and incompatible P3 amplitudes than in adults with ADHD (*p* = 0.01) (**Figure 4B**).

### Response-Locked ERPs

#### ERN

Clear ERNs were seen for erroneous responses (correctness *F*_1,51_ = 25.68, *p* < 0.001, ${\text{η}}_{\text{p}}^{2}$ = 0.33), and there was a main effect of group (diagnosis *F*_1,51_ = 4.60, *p* = 0.04, ${\text{η}}_{\text{p}}^{2}$ = 0.08). An additional interaction effect indicated that this effect was largest in controls (correctness ^∗^ diagnosis *F*_1,51_ = 4.23, *p* = 0.04, ${\text{η}}_{\text{p}}^{2}$ = 0.08), who had larger ERN amplitudes when compared to participants with ADHD (*p* < 0.001). Amplitudes were larger with overall faster RTs (RT *F*_1,51_ = 17.21, *p* < 0.001, ${\text{η}}_{\text{p}}^{2}$ = 0.25), particularly in erroneous trials (correctness ^∗^ RT *F*_1,51_ = 20.04, *p* < 0.001, ${\text{η}}_{\text{p}}^{2}$ = 0.28, *r* = 0.53) while amplitudes did not differ with age (age *F*_1,51_ = 1.52, n.s.) (**Figure 4C**).

#### Pe

The mean amplitude of the Pe component estimated for error trials was much larger than for correct responses (correctness *F*_1,51_ = 33.95, *p* < 0.001, ${\text{η}}_{\text{p}}^{2}$ = 0.4), and this effect was most pronounced in controls in erroneous trials (correctness ^∗^ diagnosis *F*_1,51_ = 5.78, *p* = 0.02, ${\text{η}}_{\text{p}}^{2}$ = 0.1). Erroneous component amplitudes were reduced with age (correctness ^∗^ age *F*_1,51_ = 4.16, *p* < 0.05, ${\text{η}}_{\text{p}}^{2}$ = 0.08). Moreover, component amplitudes were higher with faster RTs (RT *F*_1,51_ = 21.15, *p* < 0.001, ${\text{η}}_{\text{p}}^{2}$ = 0.29) for both correct (*r* = -0.36) and erroneous (*r* = -0.59) trials (correctness ^∗^ RT *F*_1,51_ = 12.8, *p* < 0.001, ${\text{η}}_{\text{p}}^{2}$ = 0.2) (**Figure 4D**).

### Correlations With Symptom Scores

**FIGURE 5:**
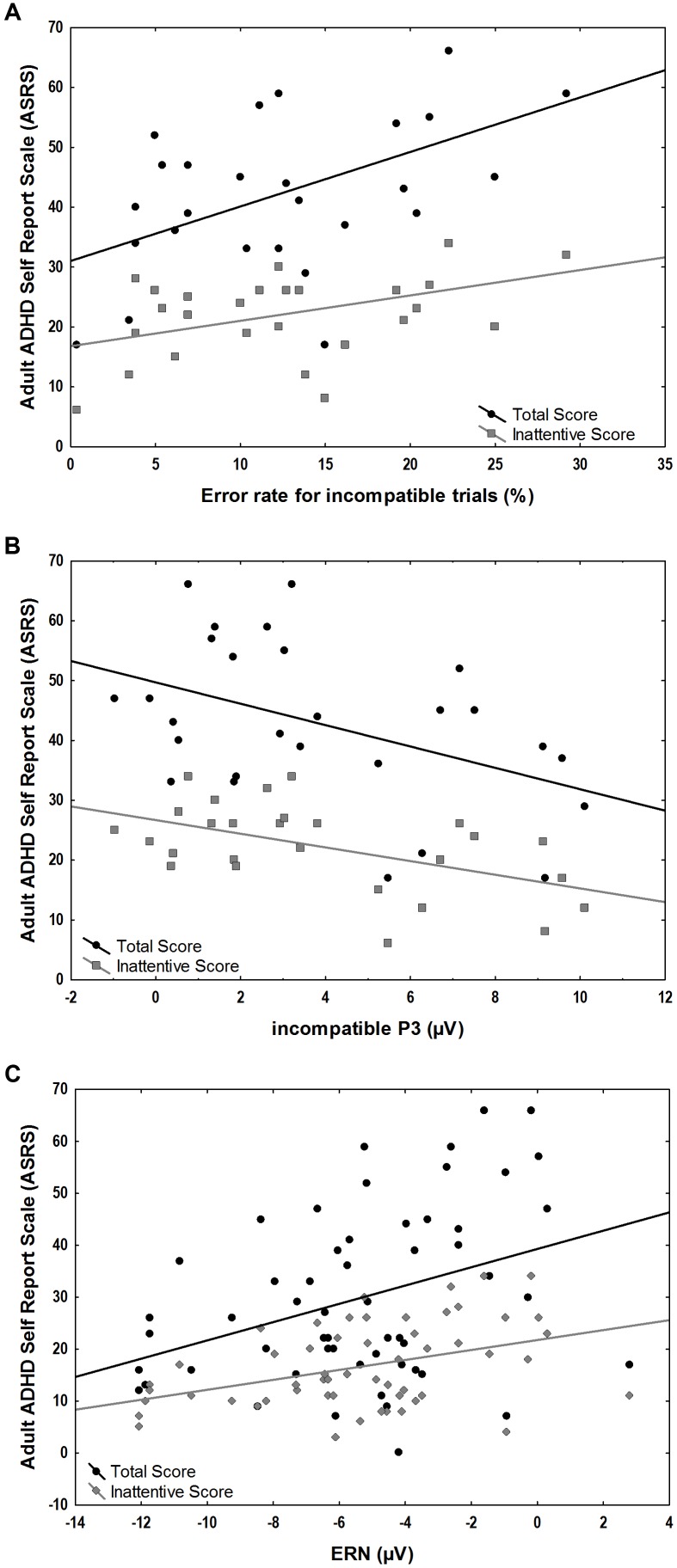
Associations between ASRS scores and ERP amplitudes and behavioral measures in the ADHD group. **(A)** Positive correlations of error-rates for incompatible trials and total (*r* = 0.52, *p* = 0.006) and inattentive ASRS scores (*r* = 0.44, *p* = 0.02). **(B)** Negative correlation of incompatible P3 correlated with total (*r* = –0.44, *p* = 0.02) and inattentive ASRS scores (*r* = –0.52, *p* = 0.005). **(C)** Positive correlation of ERN and total (*r* = 0.44, *p* = 0.02) and inattentive ASRS scores (*r* = 0.40, *p* = 0.04).

We explored the correlation of ASRS total scores and ASRS inattentive sub-scores with behavioral measures of error rates as well as with stimulus-locked incompatible P3 and response-locked component amplitudes within the ADHD group. A significant positive correlation was found between the ASRS scores and error rates in incompatible trials (total scores *r* = 0.52, *p* = 0.006, inattentive scores *r* = 0.44, *p* = 0.02) and the response-locked ERN (total scores *r* = 0.44, *p* = 0.02, inattentive scores *r* = 0.40, *p* = 0.04). Significant negative correlations were found between ASRS scores and the stimulus-locked incompatible P3 (total scores *r* = -0.44, *p* = 0.02, inattentive scores *r* = -0.52, *p* = 0.005) (**Figure 5**).

## Discussion

Here, we investigated aspects of performance monitoring in a forced-choice flanker task with speed instructions in adults with ADHD and focused on behavioral and electrophysiological indices of stimulus and error processing as well as associations with symptom load.

Addressing some inconsistencies in the available literature, our data replicate and add further evidence of behavioral and electrophysiological changes in performance monitoring in adults with ADHD. Results from P3, ERN, and Pe suggest persistent alterations of attentional and error-monitoring processes, in particular when minimizing within-subject and between-subject variability with a speeded self-paced task design, and additionally statistically controlling for confounding effects of age and response speed. Of note, we found that ERP component amplitudes and behavioral ACC correlated with ASRS scores, thus further suggesting a correspondence between electrophysiological measures and overt clinical symptom load. We discuss these results in detail in the following sections.

Behavioral performance as indicated by ACC was generally impaired in participants with ADHD, and this was especially noticeable in incompatible trials. In the modified Eriksen-flanker task used in our study, flanking arrows appeared before a central target arrow. Thus, while compatible trials can be responded quickly, a prepotent motor response caused by the appearance of the opposing flanking arrows had to be inhibited and overwritten in the incompatible condition. This process of overriding the incorrect prepotent activation that requires attentional control seems to be impaired in adults with ADHD and may explain failures to execute control in a more demanding incongruent condition (Michelini et al., 2016). Descriptively, the ADHD group also tended to respond more impulsively with higher RT variability in both compatible and incompatible trials and with slower RTs in incompatible trials than the control group. However, the differences were not significant, indicating that the temporal characteristics of performance monitoring was similar in the two groups. The absence of a significant group difference in RT and RT variability ran counter to our initial expectations and the findings of some studies (Klein et al., 2006; Chang et al., 2009; Herrmann et al., 2010; Geburek et al., 2013), but are in line with results of others (Vaidya et al., 2005; van Meel et al., 2007; Bluschke et al., 2016). The adaptive feedback procedure used in this version of the flanker task emphasizes maintaining speeded responses and might thus influence and equalize speed and variability. Given that in tasks with a slow and a speeded condition, a higher event rate as well as motivation or arousals can lead to greater improvement in RT and RT variability for ADHD participants than controls (Andreou et al., 2007; Kuntsi et al., 2009, 2013; Cheung et al., 2017), these findings may indicate that the speeded forced-choice flanker task and the given feedback used in the present study may have contributed as motivational factors to keep up speed and ACC and thereby influence energetic and motivational state regulation in adults with ADHD (Sergeant, 2005) and the malleability of RT variability. Further, ACC in incompatible trials as well as RT variability in compatible trials correlated positively with symptom load in the ADHD group in our study, indicating that patients with higher symptom loads experience more problems to regulate their attention and energetic and motivational states.

Usually, N2 is enhanced in conditions with higher conflict (Kopp et al., 1996). In our data, the difference in amplitudes between high stimulus conflict in incompatible trials and low conflict in compatible trials for both groups was not different, when controlling for age and RT. These results may be consistent with the view that the N2 reflects conflict arising from competition between the execution and the inhibition of a response and that the interference has been found to be reduced under conditions of frequent cognitive conflict like the probabilities used in our task (Nieuwenhuis et al., 2003; Folstein and Van Petten, 2008; Purmann et al., 2009; Ullsperger et al., 2014a). No amplitude differences between the groups were found, indicating that neurophysiological conflict monitoring in this task was not sensitive to problems associated with ADHD, at least after controlling for RTs (Grinband et al., 2011a,b).

In the subsequent P3 on the other hand, group differences were seen in incompatible trials, with lower amplitudes in ADHD than in the control group. This is in line with studies finding diminished P3 amplitudes in children with ADHD (Johnstone et al., 2009, 2010; Wild-Wall et al., 2009; Kratz et al., 2011; Eichele et al., 2016, 2017) and adults (McLoughlin et al., 2010, 2011; Fisher et al., 2011; Szuromi et al., 2011). We saw no differences between groups in the compatible condition, indicating either specificity to levels of conflict, or more generally scaling of function with task demand (and hence a floor effect). Importantly, voltage differences were more pronounced with higher ASRS scores, and it is therefore tempting to assume that the P3 effect corresponds to altered attentional function in adults with ADHD. The attenuation of the fronto-central P3 may in principle indicate reduced attentional orienting to incompatible stimuli, and a deficit in attentional resource allocation in demanding conditions (Brandeis et al., 2002; Lawrence et al., 2005; Johnstone et al., 2010; Kratz et al., 2011), especially in participants with high clinical symptom load.

The performance monitoring system provides signals for a need of adjustment after an error occurred, with a typical pattern of an increased negative deflection (ERN) immediately after erroneous responses that are followed by a subsequent increased positivity (Pe). The ERN was larger for controls than for adults with ADHD. This is in line with a recent meta-analysis comparing ADHD with controls and reporting an overall attenuation of ERN in performance-monitoring tasks (Geburek et al., 2013). The attenuation of the ENR in adults with ADHD may represent suboptimal error-specific early attentional processes that are connected to automatic error detection (Falkenstein et al., 1991; Gehring et al., 1993) in the fronto-striatal networks. While we provide no source localization, we assume the principal source of the ERN to be in the anterior cingulate cortex (Ullsperger et al., 2014a). This region is part of these networks, and it has been reported that people with ADHD have a smaller anterior cingulate cortex (Kasparek et al., 2015). Also, studies of individuals with ADHD have shown reduced hemodynamic activity and altered neurotransmitter levels in the anterior cingulate cortex when compared to healthy controls (Bush et al., 1999; Dramsdahl et al., 2011), and a smaller ERN in ADHD than in controls in the present study may be consistent with these findings. This is further supported by the finding of reduced ERN amplitudes with increased ASRS scores. On the other hand, ERN is known to decrease with increasing error rate (Falkenstein et al., 2000), and Ullsperger (2006) found no ERN effects in a sample of ADHD when correcting for error rate. Of note, erroneous trials showed a sustained negativity that starts deviating already 600 ms before response onset (**Figures 2C,D**), suggesting that (premature) error commission indeed starts early during stimulus processing (Eichele et al., 2010) and may reflect less active subprocesses or modulations in adults with ADHD.

After the early error detection, reflected by the ERN, the following stage of error processing, reflected by the Pe, also appeared to be hampered in adults with ADHD in our study. While the ERN was found to be present on both recognized and unrecognized errors, the Pe was present only in trials on which the subjects were aware of their errors (Nieuwenhuis et al., 2001). Based on this, the Pe may reflect evaluation of the error response and its motivational significance, along with the initiation of adaptive control processes, while the lower Pe amplitude may point at reduced error awareness and conscious error processing in the ADHD group. A recent meta-analysis with adult ADHD patients found that the pooled effect size for the Pe was significantly reduced for the Go-NoGo task but not for the flanker task (Geburek et al., 2013). They interpreted that motor inhibition after an error in the Go-NoGo task is easily perceived for the controls in contrast to the ADHD group, while both groups struggled with the more complex design of a flanker task and hence leading to less pronounced differences in Pe amplitudes between the groups (Geburek et al., 2013). However, the flanker task used in our study provides feedback after errors which could generally have influenced response caution in the control group and thereby helped controls to improve behavioral and electrophysiological performance monitoring. In contrast, adults with ADHD may have adapted their behavior to a certain degree, which is reflected in non-significant speed and variability differences between the controls and the ADHD group, although still not compensated sufficiently for, as seen in increased error rates. However, on the electrophysiological level, the underlying processes of performance monitoring do not seem to be accessible to conscious compensation efforts, as seen in attenuation of ERP measures.

Among the limitations of our study, the relatively small sample size should be mentioned, which also limited the possibility to analyze the effect of comorbidities, gender, and use of medication. Ideally, the impact of comorbid conditions and medication should be assessed separately, and in more detail, however, we performed exploratory analyses between these subsamples in the dependent measures and did not find any significant differences between the subsamples. Furthermore, the ADHD patients recruited in this study were relatively well-functioning, making it difficult to transfer the results to other more impaired clinical samples (Lundervold et al., 2015).

## Conclusion

Taken together, the present examination of cognitive processes related to performance monitoring in adults with ADHD adds to our knowledge of electrophysiological correlates of monitoring and response control deficits in adults with ADHD. Adults with ADHD show attenuated P3, ERN, and Pe, which points to an impairment of attentional resource allocation in demanding conditions, automatic error detection, as well as error awareness. Behavioral performance measures were not significantly different between the groups, while group differences were more readily seen in ERP measures. This suggests that the ERP measures are even more sensitive to the underlying liability for ADHD than the overt behavioral task performance. Future work on ADHD might benefit from focusing on these early processes of performance and error monitoring to further delineate the pathogenesis of ADHD. Such results may aid in development of intervention strategies, especially targeting the increased requirements of self-management in the life of adult ADHD patients.

## Author Contributions

HE, JH, AL, and TE were involved in the conception and design of the work. HE, LM, and TE were involved in the acquisition of the data. HE, LM, and TE were involved in the analysis and interpretation of data and writing of the article. HE, LM, AL, JH, and TE were involved in the critical review of article and agreement to be accountable for all aspects of the work.

## Conflict of Interest Statement

JH has served as a speaker for Eli-Lilly, HB Pharma, and Shire. The other authors declare that the research was conducted in the absence of any commercial or financial relationships that could be construed as a potential conflict of interest.
